# Benchmarking Multimodal Deep Fusion Strategies for Heterogeneous Neuroimaging and Cognitive Data Using a Controlled Sex Classification Task

**DOI:** 10.3390/brainsci16040405

**Published:** 2026-04-10

**Authors:** Chiara Camastra, Assunta Pelagi, Andrea Quattrone, Alessia Sarica

**Affiliations:** 1 Department of Medical and Surgical Sciences, Magna Graecia University, 88100 Catanzaro, Italy; chiara.camastra@unicz.it (C.C.); assunta.pelagi@studenti.unicz.it (A.P.); 2 Brain Health Imaging Centre, Centre for Addiction and Mental Health, Toronto, ON M6J 1H4, Canada; 3 Neuroscience Research Center, Magna Graecia University, 88100 Catanzaro, Italy; an.quattrone@unicz.it; 4 Institute of Neurology, Department of Medical and Surgical Sciences, Magna Graecia University, 88100 Catanzaro, Italy

**Keywords:** multimodal data fusion, heterogeneous data, early fusion, intermediate fusion, late fusion, feature scaling, human connectome project

## Abstract

**Highlights:**

**What are the main findings?**

- Early feature-level fusion consistently outperforms intermediate and late fusion strategies in multimodal sex classification.
- Standard feature scaling significantly enhances multimodal deep learning performance across architectures.

**What are the implications of the main findings?**

- Architectural complexity does not guarantee superior performance in heterogeneous multimodal integration.
- Fusion strategy and preprocessing must be jointly optimized for reliable and reproducible multimodal modeling in neuroscience.

**Abstract:**

**Background/Objectives**: Multimodal data fusion is increasingly applied in neuroinformatics to integrate heterogeneous sources of information. However, the optimal strategies for combining modalities with markedly different dimensionality, scale, and noise characteristics remain unclear. To our knowledge, this is among the first systematic and controlled benchmarks explicitly disentangling the effects of fusion strategy and feature scaling within a unified deep learning framework. **Methods**: Using data from 747 healthy participants from the Human Connectome Project, we evaluated multiple fusion paradigms—including early fusion, attention-based fusion, subspace-based fusion, and graph-based fusion—within a unified and reproducible framework. Importantly, we assessed how different feature scaling techniques (Standard, Min–Max, and Robust scaling) interact with fusion strategies and influence model performance. Biological sex was used as a controlled benchmark task to focus on methodological insights rather than task-specific optimization. **Results**: Early feature-level fusion consistently achieved the highest classification performance across all evaluated configurations. In particular, direct concatenation of cognitive and neuroimaging features combined with Standard Scaling yielded the best results (AUC–ROC = 0.96 (0.95–0.96)), outperforming unimodal baselines as well as intermediate and late fusion strategies. **Conclusions**: This systematic benchmark demonstrates that multimodal deep learning performance in neuroscience is driven primarily by the interaction between fusion strategy and feature scaling rather than by architectural complexity alone. By explicitly disentangling the effects of fusion level and preprocessing within a unified framework, this study provides practical methodological guidance for the design, evaluation, and reproducible deployment of multimodal deep learning models in neuroscience.

## 1. Introduction

Understanding brain organization and behavior increasingly relies on the integration of heterogeneous data sources, including neuroimaging, cognitive, and clinical measures [1]. These modalities provide complementary perspectives on brain structure and function but differ substantially in dimensionality, scale, noise characteristics, and statistical distribution [2,3]. Neuroimaging-derived features are typically high-dimensional and spatially correlated [4], whereas cognitive or clinical variables are low-dimensional, behaviorally defined, and often affected by skewed distributions, ceiling effects, or outliers [5]. This heterogeneity poses significant challenges for joint modeling and interpretation, thereby motivating the development of computational strategies capable of integrating complementary information across modalities.

In this context, multimodal data fusion has emerged as a central strategy for combining heterogeneous information sources within a single modeling framework. Across data-driven modeling approaches—including statistical learning, signal processing, and machine learning—the effective fusion of complementary modalities has been shown to improve model accuracy, robustness, and generalization, whereas suboptimal fusion strategies can lead to degraded performance, instability, and overfitting [6]. As a result, the choice of fusion strategy is now recognized as a critical component of model design rather than a secondary implementation detail [7].

In neuroscience and biomedical research, multimodal fusion strategies are commonly categorized into early (feature-level), intermediate (representation-level), and late (decision-level) approaches [8]. Early fusion combines modalities by concatenating features prior to model learning, intermediate fusion integrates learned representations through shared latent spaces or attention mechanisms, and late fusion aggregates predictions from separate unimodal models [9]. Recent advances in deep learning–based multimodal fusion have introduced increasingly expressive architectures—such as attention-based models, variational subspace learning, and graph neural networks (GNN)—that are specifically designed to address modality heterogeneity and complex cross-modal interactions [6,10,11].

The relevance of deep learning–based multimodal data fusion has been demonstrated across several areas of neuroscience and medicine [12]. In oncology and precision health, integrating heterogeneous data types has improved predictive modeling and facilitated biomarker discovery [13,14]. Similar challenges arise in neuroscience, where comprehensive characterization of brain organization and disease often requires combining neuroimaging with clinical, cognitive, genetic, or demographic variables [15,16,17]. In neurodegenerative disease research, for example, multimodal approaches have enabled the identification of composite biomarkers linking brain degeneration to molecular and genetic factors [18,19,20,21,22,23]. Despite this growing body of work, many studies primarily focus on maximizing predictive performance, with limited attention to the methodological trade-offs associated with fusion strategy selection and preprocessing choices. In particular, recent advances have introduced increasingly complex multimodal architectures—such as attention-based models, variational autoencoders, and graph neural networks—designed to capture cross-modal interactions and improve representation learning [6,24,25,26]. While these approaches have shown promising results [9,27], direct comparisons across fusion strategies are often lacking, and their performance is rarely evaluated within a unified and controlled experimental framework.

Consequently, it remains unclear whether increased architectural complexity consistently translates into improved performance [28] or whether simpler fusion strategies may be equally or more effective under specific data conditions. Moreover, the interaction between fusion strategy and preprocessing—particularly feature scaling—has been largely overlooked, despite its potential impact on model stability and generalization. Addressing these limitations requires systematic benchmarking studies that disentangle the contribution of fusion strategy and preprocessing within a consistent and reproducible framework.

Within this methodological context, biological sex represents a well-characterized and biologically grounded reference variable for benchmarking multimodal learning approaches in neuroscience. Sex-related differences in brain structure and function have been consistently reported across large-scale neuroimaging studies, involving both global and regional measures such as total brain volume, cortical thickness, white matter microstructure, and functional connectivity [29,30,31,32]. In parallel, cognitive differences between males and females have also been described across multiple domains, including working memory, verbal fluency, spatial reasoning, and emotional recognition [33,34,35,36]. Importantly, the use of sex classification in this study was not motivated by the investigation of biological sex itself, but rather by the availability of a stable and reproducible task with well-established signal properties. In this setting, classification performance primarily reflects the ability of deep-learning models to integrate heterogeneous information sources rather than ambiguity or uncertainty in the target variable.

In this study, we systematically evaluated deep learning–based unimodal and multimodal fusion strategies to investigate how different approaches to data integration influence model performance when combining neuroimaging and cognitive information. Imaging-derived brain morphology metrics were integrated with cognitive test scores from 747 healthy participants from the Human Connectome Project (HCP) Young Adult dataset, with biological sex serving as a controlled benchmark variable. Cognitive features were intentionally included to introduce a low-dimensional and heterogeneous modality, thereby reflecting realistic neuroscience research scenarios rather than maximizing classification performance. Within this framework, multiple fusion paradigms—including early, attention-based, subspace-based, and graph-based approaches—were compared under controlled experimental conditions using a unified and reproducible pipeline implemented in the *Fusilli* Python library (version 3.9). We further assessed the impact of different feature scaling strategies (Standard, Min–Max, and Robust scaling) on model performance and robustness. Overall, this work provides a systematic methodological benchmark and a practical reference for the design and evaluation of multimodal deep learning models in neuroscience.

The contributions of this study can be summarized as follows:

(i) We provide a systematic and controlled benchmark of multimodal deep learning fusion strategies, comparing early, intermediate, and late fusion approaches within a unified experimental framework;
(ii) We explicitly investigate the impact of feature scaling on multimodal model performance, highlighting its critical role in heterogeneous data integration;
(iii) We implement and evaluate all models within a consistent and reproducible pipeline, enabling fair comparison across architectures;
(iv) We provide practical insights into the design of multimodal learning systems for neuroscience applications.

## 2. Materials and Methods

### 2.1. Data Preparation

The WU-Minn HCP Young Adult dataset, comprising 1200 healthy subjects (including adult twins and their non-twin siblings, aged 22–36), was selected for this study [37]. Restricted-access HCP data tables—providing detailed information on family structure (e.g., twin status), age, handedness, ethnicity, and race—were downloaded from the ConnectomeDB database on 11 December 2023 (https://www.humanconnectome.org/study/hcp-young-adult/document/wu-minn-hcp-consortium-open-access-data-use-terms). Dataset preprocessing was performed using a custom workflow implemented in the KNIME Analytics Platform (version 4.6.1) [38]. To reduce potential confounding effects on brain morphology, participants who tested positive for drugs or alcohol were excluded. In addition, to ensure demographic consistency and reduce population-related variability, only participants who self-identified as White or Black were included in the analysis. Following preprocessing and quality control, the final study cohort comprised 747 participants (410 females and 337 males). From the processed data, two separate feature matrices were constructed: one containing neuroimaging-derived features and one containing cognitive and behavioral measures.

The feature set included the following two categories.

**(1) Cognitive Features**

The cognitive dataset comprised 36 cognitive and behavioral measures spanning multiple domains, including executive function, working memory, emotional processing, impulsivity, sleep quality, and personality traits. Key assessments included the Card Sorting Task, which evaluates cognitive flexibility; the Flanker Task, assessing attentional control and response inhibition; the Picture Sequence Memory Test, measuring episodic memory; and the Delay Discounting Task, capturing impulsivity and decision-making behavior.

Additional measures included personality trait scores for Neuroticism, Openness, and Conscientiousness; the Pittsburgh Sleep Quality Index (PSQI), assessing sleep quality; the Emotion Recognition Task (ER40), evaluating emotion recognition; and the Adult Self-Report (ASR) Anxiety score, reflecting emotional and psychological well-being.

**(2) Neuroimaging Features**

Neuroimaging-derived features were obtained using the FreeSurfer image analysis suite and consisted of 168 brain morphology metrics, including volumetric, cortical thickness, and cortical surface area measures. These features encompassed subcortical volumes (e.g., thalamus, caudate, putamen, hippocampus), ventricular volumes, cerebellar structures, as well as regional cortical thickness and surface area measurements across multiple cortical regions (e.g., superior frontal, supramarginal, and insular cortices).

The final input data matrix consisted of 747 samples and 204 features (36 cognitive and 168 neuroimaging features). Therefore, the dimensionality of the multimodal input was 747 × 204, resulting in a feature-to-sample ratio of approximately 0.27.

### 2.2. Data Fusion Workflow

**Figure 1:**
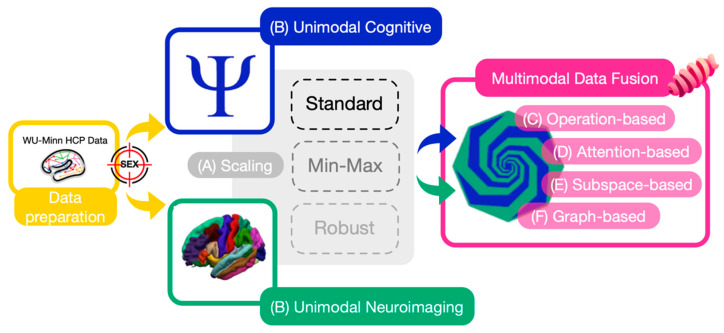
Proposed workflow for multimodal sex classification using cognitive and neuroimaging features. The pipeline integrates data from the WU–Minn Human Connectome Project (HCP) and is structured into three main stages: (A) feature scaling, (B) unimodal processing of cognitive and neuroimaging modalities, and (C–F) multimodal data fusion. Four fusion strategies are evaluated: (C) operation-based, (D) attention-based, (E) subspace-based, and (F) graph-based approaches.

An overview of the multimodal data fusion workflow used in this study is shown in Figure 1. The pipeline is structured into three main stages: (A) feature scaling, (B) unimodal processing, and (C) multimodal fusion.

The data preparation phase (Figure 1, yellow box) involves the selection and preprocessing of features from the WU-Minn HCP Young Adult dataset, including imaging-derived brain morphology metrics and cognitive test scores. The classification target is biological sex (male vs. female).

In the unimodal processing stage (Figure 1, blue and green boxes), cognitive and neuroimaging features are processed separately. Each modality undergoes feature scaling (Figure 1, gray box), where three common normalization techniques—Standard Scaling, Min–Max Scaling, and Robust Scaling—are systematically compared to evaluate their impact on model performance and data integration.

The multimodal fusion stage (Figure 1, pink box) integrates the two modalities using four deep learning-based fusion strategies implemented in the *Fusilli* v1.2.3 Python library [39]. Specifically, the following approaches are evaluated:

- Operation-based fusion (e.g., feature concatenation);
- Attention-based fusion (e.g., multi-head attention mechanisms);
- Subspace-based fusion (e.g., variational autoencoders);
- Graph-based fusion (e.g., graph neural networks).

All models were implemented in Python 3.10 using *Fusilli*, a library built on PyTorch (version 2.7.1) and PyTorch Lightning (version 2.6.1) that facilitates the design, training, and comparison of multimodal fusion architectures.

Prior to model training, the dataset was formatted to meet *Fusilli*’s input specifications using the prepare_fusion_data() utility. This required each sample to include a unique identifier and a prediction_label column with integer-coded targets. The classification task was therefore defined as a binary prediction problem aimed at distinguishing male and female participants based on integrated cognitive and neuroanatomical features. Each step of the pipeline is described in detail in the following sections.

No explicit feature selection or dimensionality reduction was applied prior to model training. This choice was intentional, as the aim of this study was to benchmark multimodal fusion strategies under controlled conditions, ensuring that all models received the same input information. Furthermore, deep learning models inherently perform implicit feature selection through weight optimization during training.

#### 2.2.1. Data Scaling

Three widely used feature scaling techniques were evaluated: Standard Scaling, Min–Max Scaling, and Robust Scaling, each designed to address different data distribution characteristics (Figure 1A) [40].

Standard Scaling normalizes each feature to have zero mean and unit variance. This transformation is defined as:

$$X_{\mathrm{scaled}}\;=\;\frac{X-\mu }{\sigma }$$

where X is the original feature value, μ is the mean, and σ is the standard deviation.

This method is particularly effective for approximately normally distributed data; however, it is sensitive to the presence of outliers.

Min–Max Scaling, in contrast, rescales features to a fixed range, typically [0, 1], making it suitable for non-normally distributed data without extreme outliers. The transformation is computed as:

$$X_{\mathrm{scaled}}\;=\;\frac{X-Xmin}{Xmax-Xmin}$$

where X denotes the original feature value, and X_min_ and X_max_ are the minimum and maximum values of the feature, respectively. Because this method relies on the observed minimum and maximum, it is particularly sensitive to outliers.

Finally, Robust Scaling is specifically designed to reduce the influence of outliers by scaling features based on the median and the interquartile range (IQR), rather than the mean and standard deviation. The transformation is given by:

$$X_{\mathrm{scaled}}\;=\;\frac{X-Median(X)}{IQR(X)}$$

where X represents the original feature value, Median(X) is the median of the feature, and IQR(X) is the interquartile range, calculated as Q_3_ − Q_1_ (with $Q_{3}$ and $Q_{1}$ denoting the third and first quartiles, respectively). This approach is particularly effective for datasets with skewed distributions or prominent outliers, as it minimizes their influence on the scaling process.

#### 2.2.2. Deep Learning Models for Unimodal and Multimodal Fusion

##### Unimodal Deep Learning Models

Before multimodal integration, two unimodal deep learning models were trained and evaluated to establish baseline predictive performance (Figure 1B). Tabular Unimodal 1 corresponds to the cognitive feature set, whereas Tabular Unimodal 2 corresponds to the neuroimaging-derived brain morphology features. Both unimodal models employ fully connected neural networks to process each modality independently, providing a reference for the predictive contribution of each data source prior to multimodal fusion.

##### Early Fusion Models (Feature-Level Fusion)

Early fusion models integrate information from multiple modalities at the feature level, prior to learning higher-level representations. In these approaches, modality-specific features or feature maps are combined directly and processed jointly by downstream neural network layers.

- **Concat Tabular Data.**

In this model, the raw features from the cognitive and neuroimaging modalities are concatenated into a single feature vector, which is subsequently processed through fully connected layers to generate the final prediction.

- **Concat Tabular Feature Maps.**

Each tabular modality is first processed independently through its own fully connected layers to obtain modality-specific feature maps. These feature representations are then concatenated and passed through additional fully connected layers to produce the final prediction.

- **Channel-Wise Multi Net.**

In this early fusion architecture, modality-specific feature representations are combined using channel-wise weighting mechanisms, allowing the model to emphasize or attenuate feature channels from each modality before classification. The fused representation is subsequently processed by fully connected layers to produce the final output.

##### Intermediate Fusion Models (Representation-Level Fusion)

Intermediate fusion models integrate information across modalities after learning modality-specific representations, enabling interaction between higher-level features through shared latent spaces, attention mechanisms, or graph-based structures.

**Attention-based fusion models**

Attention-based models represent a specific class of intermediate fusion approaches in which cross-modal integration is achieved by explicitly learning adaptive weighting schemes over modality-specific representations [41,42]. Through self- and cross-attention mechanisms, these models dynamically modulate the contribution of each modality, allowing the network to prioritize the most informative features during multimodal integration.

- **Tabular Crossmodal Multihead Attention.**

Each tabular modality is first processed independently through fully connected layers. Self-attention is applied within each modality, followed by cross-modal multihead attention to enable interaction between modalities. The output of the cross-modal attention module is then passed through a fully connected layer to generate the final prediction. This model is inspired by the Multimodal Attention Deep Learning Framework (MADDi) framework proposed by Golovankesky et al. [21].

- **Activation Function and Tabular Self-Attention.**

This model combines self-attention mechanisms applied to tabular feature representations with nonlinear activation-based fusion, enabling the network to capture complex cross-modal interactions at the representation level before classification.

**Subspace-based fusion models**

Subspace-based models aim to learn a shared latent representation that captures common information across modalities.

- **MCVAE Tabular.**

This model adopts the Multi-channel Variational Autoencoder (MCVAE) framework introduced by Antelmi et al. [26]. Each tabular modality is treated as a separate channel and processed through a variational autoencoder with a modified loss function, enabling the learning of a joint latent space shared across modalities. The resulting one-dimensional latent representation is subsequently processed through fully connected layers to generate the final prediction [25].

**Graph-based fusion models**

Graph-based fusion models represent subjects as nodes in a graph, where edges encode similarity relationships derived from multimodal features [26,43].

- **Edge Correlation GNN.**

This tabular–tabular model constructs a graph by computing correlations between features of the first tabular modality. Correlation values are used as edge weights, and edges are removed if the correlation falls below a predefined threshold (default = 0.8). Features from the second tabular modality are used as node attributes. The resulting graph is processed through a graph neural network to generate predictions.

- **Attention-Weighted GNN.**

Inspired by the population-graph learning approach proposed by Bintsi et al. [27], this model employs a static graph representation. The model is pretrained using the Concat Tabular Data fusion architecture to learn attention weights. These weights are then applied to the concatenated tabular features. Pairwise Euclidean distances between subjects are computed, and edges are created between subjects whose distances fall within the lowest 25% of the distribution. The resulting graph is processed through a graph neural network to generate the final prediction.

##### Late Fusion Models (Decision-Level Fusion)

Late fusion models combine information at the decision level by aggregating predictions from modality-specific models.

- **Tabular Decision.**

In this approach, each tabular modality is processed independently through its own fully connected neural network, producing separate predictions. The final output is computed as the average of the modality-specific predictions, implementing a decision-level fusion strategy.

#### 2.2.3. Hyperparameter Tuning and Performance Evaluation

For hyperparameters shared across all models, the batch size was set to 64, the maximum number of training epochs to 10,000, and the learning rate to 1 × 10^−6^. Binary classification performance was evaluated using 3-fold cross-validation, and results were reported in terms of mean area under the receiver operating characteristic curve (AUC–ROC) and accuracy. In addition to summary metrics, ROC curves and precision–recall (PR) curves were computed to assess model performance across different decision thresholds. Furthermore, ROC curves were specifically analyzed in the low false-positive rate regime to evaluate classifier performance under stringent operating conditions.

Complementary performance metrics were also reported, including balanced accuracy, F1-score, Cohen’s kappa, precision, recall, and average precision, to provide a more comprehensive evaluation of classification performance.

To quantify the uncertainty of model performance estimates, 95% confidence intervals (CI) were computed for all evaluation metrics using bootstrap resampling of fold-wise results.

Overfitting was monitored through continuous inspection of learning curves for all models. Specifically, overfitting was defined as a consistent and persistent divergence between training and validation performance, such as decreasing training loss without a corresponding improvement—or with a deterioration—of validation metrics. When such behavior was observed, predefined corrective strategies were applied, including simplification of model architectures, adjustment of the depth and width of fully connected layers, introduction of dropout layers, and revision of preprocessing steps.

For models supporting early stopping mechanisms (e.g., attention-based architectures), validation performance was monitored using a patience parameter set to 250 epochs. Although a high maximum epoch limit (10,000) was adopted to allow models to fully converge, early stopping enabled automatic termination of training once performance stabilized, thereby mitigating overfitting.

For models that did not support automatic early stopping, learning curves were inspected post hoc to verify that overfitting had not occurred despite full-length training. When evidence of overfitting was identified, hyperparameters were re-tuned accordingly (Table 1). This iterative process of monitoring and adjustment ensured that the final models were trained under conditions that minimized overfitting while maximizing generalization performance across modalities.

The overall workflow of the proposed framework is summarized in Algorithm 1.

### 2.3. Pseudo-Code of the Proposed Framework

To provide a clear and structured overview of the proposed multimodal fusion pipeline, we summarize the main processing steps in Algorithm 1. The pseudo-code outlines the key stages of the framework, including data preprocessing, feature scaling, unimodal modeling, multimodal fusion strategies, and performance evaluation.
**Algorithm 1.** Multimodal Fusion PipelineInput: Cognitive features Xc, Imaging features Xi, Labels y 1. Preprocess data     - Clean dataset     - Split into folds (cross-validation) 2. Apply feature scaling     For each scaling method in {Standard, Min–Max, Robust}:          - Xc_scaled ← scale(Xc)          - Xi_scaled ← scale(Xi) 3. Unimodal modeling     - Train model Mc on Xc_scaled     - Train model Mi on Xi_scaled 4. Multimodal fusion     For each fusion strategy:          (a) Early fusion:                X_fused ← concatenate(Xc_scaled, Xi_scaled)                Train model M_fused on X_fused          (b) Intermediate fusion:                hc ← encoder_c(Xc_scaled)                hi ← encoder_i(Xi_scaled)                h_fused ← fusion_module(hc, hi)                Train classifier on h_fused          (c) Late fusion:                yc ← Mc(Xc_scaled)                yi ← Mi(Xi_scaled)                y_fused ← combine(yc, yi) 5. Model training     - Optimize parameters     - Apply early stopping when available 6. Evaluation     - Compute AUC-ROC, Accuracy, Balanced Accuracy, F1-score, Cohen’s kappa, Average Precision     - Average results across folds Output: Performance metrics for each fusion strategy

## 3. Results

The final study cohort comprised 747 healthy participants from the HCP Young Adult dataset, including 410 females and 337 males (Table 2). Participants were aged between 22 and 36 years and showed a highly homogeneous cognitive profile, as expected for a healthy young adult population.

Demographic characteristics were largely comparable between female and male participants, with similar years of education and Mini-Mental State Examination (MMSE) scores. Females were slightly older on average than males, although the age distributions largely overlapped between groups. Overall, the cohort was well balanced and suitable for benchmarking multimodal learning approaches under controlled conditions.

### 3.1. Unimodal Deep Learning Models

Unimodal models provided a baseline for evaluating the contribution of each modality independently. The cognitive-only model (Tabular1 Unimodal) achieved moderate classification performance across all scaling methods, with the highest performance observed under Robust Scaling (AUC–ROC = 0.78 (0.77–0.81)), consistent with the presence of outliers and broader feature distributions in cognitive measures.

In contrast, the neuroimaging-only model (Tabular2 Unimodal) consistently outperformed the cognitive model across all preprocessing strategies, reaching its highest performance under Robust Scaling (AUC–ROC = 0.93 (0.93–0.94)). This result reflects the more bounded numerical ranges and approximately Gaussian distributions of brain morphology features, which are well suited to standard normalization. Overall, unimodal results confirm the stronger discriminative signal contained in neuroimaging features while highlighting the sensitivity of cognitive features to preprocessing choices (Table 3).

### 3.2. Early Fusion Models

Early fusion approaches, which combine modalities at the feature level prior to model learning, yielded the highest overall classification performance across all evaluated configurations. Among these models, Concatenating Tabular Data consistently achieved the best performance, reaching a peak AUC–ROC of 0.96 (0.95–0.96) under Standard Scaling and maintaining strong results under Min–Max and Robust Scaling (AUC–ROC = 0.94 (0.92–0.98) and 0.95 (0.94–0.95), respectively).

Concatenating Tabular Feature Maps also performed competitively under Standard and Min–Max Scaling (AUC–ROC = 0.90 (0.89–0.92) and 0.93 (0.91–0.95), respectively), although performance degraded under Robust Scaling. The Channel-Wise Multi Net model showed lower and more variable performance, suggesting reduced robustness to scaling choices.

Overall, early fusion models benefited most from Standard Scaling, particularly when raw features were directly concatenated, indicating that early integration of modalities is highly effective when feature distributions are appropriately normalized (Table 3).

### 3.3. Intermediate Fusion Models

Intermediate fusion models, which integrate modalities after learning modality-specific representations through shared latent spaces or attention mechanisms, exhibited more heterogeneous performance patterns compared to early fusion approaches. Among these models, attention-based architectures—particularly the Tabular Crossmodal Multi-Head Attention—achieved consistently high and stable performance across all scaling strategies, with AUC–ROC values of 0.92 (0.89–0.92), 0.94 (0.93–0.96), and 0.92 (0.91–0.94) for Standard, Min–Max, and Robust Scaling, respectively. These results indicate a strong ability to adapt weight modality-specific information despite differences in feature distributions.

The Activation-Function and Tabular Self-Attention model demonstrated intermediate performance, achieving its best result under Robust Scaling (AUC–ROC = 0.80 (0.79–0.81)), highlighting a marked sensitivity to preprocessing choices.

Graph-based intermediate fusion models showed mixed behavior. The Edge Correlation GNN achieved strong performance under Standard Scaling (AUC–ROC = 0.92 (0.90–0.94)) but exhibited a notable decline under Min–Max and Robust Scaling. Conversely, the Attention-Weighted GNN consistently underperformed across all preprocessing pipelines (AUC–ROC ≈ 0.50), indicating limited suitability for this task despite adaptive graph construction (Table 3).

### 3.4. Late Fusion Models

Late fusion approaches, which combine modality-specific predictions at the decision level, demonstrated stable and competitive performance across all evaluated scaling strategies. In this category, the Tabular Decision model consistently achieved AUC–ROC values in the range of 0.91–0.93, indicating robustness to preprocessing choices and effective aggregation of unimodal predictions (Table 3).

### 3.5. Effect of Feature Scaling Across Fusion Strategies

**Figure 2:**
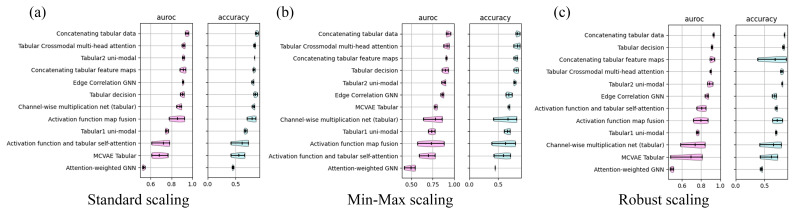
Performance comparison of unimodal and multimodal fusion models across three feature scaling strategies: (**a**) Standard Scaling, (**b**) Min–Max Scaling, and (**c**) Robust Scaling. For each scaling method, boxplots report the distribution of AUC-ROC (left) and accuracy (right) across 3-fold cross-validation. Models include unimodal baselines (Tabular1, Tabular2), early fusion approaches (e.g., Concatenating Tabular Data, Concatenating Tabular Feature Maps), intermediate fusion models (e.g., Tabular Crossmodal Multi-Head Attention, MCVAE Tabular, Edge Correlation GNN, Attention-Weighted GNN), and decision-level late fusion (Tabular Decision). Pink boxplots represent AUC-ROC, while light blue boxplots represent accuracy.

Across all model categories, Standard Scaling emerged as the most effective preprocessing strategy, particularly for early fusion and graph-based models (Figure 2a). Min–Max Scaling produced competitive results for several architectures but introduced performance instability in models sensitive to compressed feature ranges (Figure 2b). Robust Scaling proved beneficial primarily for cognitive features and selected intermediate fusion models but was generally suboptimal for high-performing early fusion approaches (Figure 2c). A comprehensive summary of model performance, including balanced accuracy, F1-score, Cohen’s kappa, and average precision, is provided in Supplementary Table S1.

**Figure 3:**
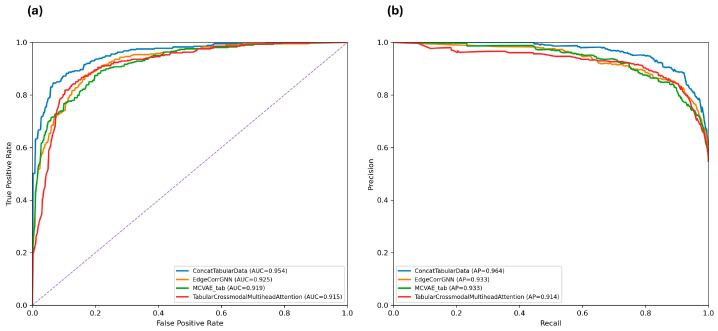
Receiver operating characteristic (ROC) and precision–recall (PR) curves for the best-performing models under Standard Scaling. (**a**) ROC curves and (**b**) PR curves are shown for selected unimodal and multimodal fusion models evaluated using 3-fold cross-validation. Curves are computed from aggregated predictions across folds, and AUC–ROC and average precision values are reported in the legend.

ROC and PR curves for the best-performing models are shown in Figure 3 for Standard Scaling, while corresponding results for Min–Max and Robust Scaling are reported in Supplementary Figure S1.

To further assess classifier behavior under stringent operating conditions, ROC curves were additionally examined in the low false-positive rate regime (Supplementary Figure S2), providing a detailed view of performance at low false alarm levels. In this regime, differences between models become more apparent. Under Standard Scaling, early fusion (Concatenating Tabular Data) maintains consistently higher true positive rates at very low false-positive levels compared to other models. In contrast, under Min–Max and Robust Scaling, the separation between models is reduced, with curves showing greater overlap and less pronounced differences in the low false-positive region.

**Figure 4:**
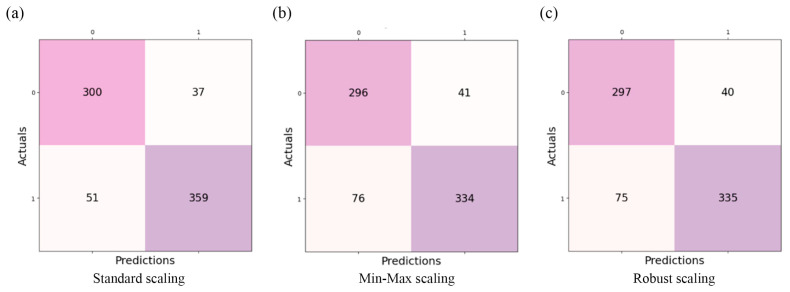
Confusion matrices for the best-performing fusion model (Concatenating Tabular Data) across three preprocessing pipelines: (**a**) Standard Scaling, (**b**) Min–Max Scaling, and (**c**) Robust Scaling. Each matrix shows the distribution of predicted versus true class labels across the test folds. Standard Scaling yields the highest classification performance (AUC-ROC = 0.96 (0.95–0.96)) and the lowest number of misclassifications.

Confusion matrices for the best-performing model (Concatenating Tabular Data) further support these findings (Figure 4), with Standard Scaling yielding the lowest misclassification rate and the highest AUC-ROC (0.96 (0.95–0.96)).

## 4. Discussion

This study provides a systematic methodological benchmark of unimodal and multimodal deep learning fusion strategies for integrating heterogeneous cognitive and neuroimaging data, using biological sex as a stable reference task. Our results show that early feature-level fusion—particularly direct concatenation of tabular data combined with Standard Scaling—consistently achieved the highest classification performance, outperforming unimodal baselines as well as more complex intermediate and late fusion approaches.

These findings are consistent with recent theoretical work comparing early and late fusion strategies in binary multimodal classification problems [44]. Such analyses indicate that early fusion is advantageous when modalities are fully observed and jointly informative, as it enables direct modeling of cross-modal feature interactions. Conversely, late fusion may be more appropriate in settings characterized by modality-specific noise, conditional independence, or incomplete data. In the present controlled setting, the superior performance of early fusion likely reflects complete data availability and consistent preprocessing across modalities. Under these conditions, direct integration allows models to capture complementary cross-modal information more effectively, whereas latent representations or decision-level aggregation may constrain such information [6,11].

Our findings can be further contextualized within the broader literature on multimodal deep learning. Recent studies have proposed increasingly sophisticated fusion architectures, including attention-based models, variational autoencoders, and graph neural networks, often reporting improved predictive performance in complex biomedical and neuroimaging tasks [6,9,45,46,47]. In particular, attention mechanisms can enhance multimodal integration by dynamically weighting modality-specific features, while graph-based approaches aim to capture inter-subject relationships and population-level structure [26].

However, these approaches are typically evaluated in more complex scenarios, involving heterogeneous populations, partially observed modalities or higher task difficulty. In contrast, the present study was conducted under controlled conditions with fully observed modalities and standardized preprocessing across all subjects. Within this setting, early fusion strategies based on direct feature concatenation achieved equal or superior performance compared to more complex architectures. These results indicate that the benefit of advanced fusion models is not universal but depends critically on the statistical properties of the data, the degree of complementarity between modalities, and the experimental context.

Importantly, these results highlight that methodological factors—particularly preprocessing and feature scaling—play a central role in determining model performance. Across all fusion levels, feature scaling emerged as a critical determinant of performance, underscoring that preprocessing should be considered an integral component of multimodal model design rather than a secondary technical step [48]. In contrast to prior work emphasizing architectural novelty or peak predictive accuracy [49,50], the present results demonstrate that early fusion strategies can outperform more expressive models when appropriately aligned with the statistical properties of the data.

Unimodal analyses further showed that imaging-derived brain morphology features provide a stronger and more stable discriminative signal than cognitive measures alone [51]. At the same time, cognitive features were more sensitive to preprocessing choices, benefiting particularly from Robust Scaling due to broader distributions and the presence of outliers. Intermediate fusion approaches exhibited more heterogeneous behavior: attention-based models showed relatively stable performance across scaling strategies, suggesting robustness to modality heterogeneity, whereas subspace-based fusion using variational autoencoders resulted in reduced discriminative power, likely due to information compression in low-dimensional latent space [52]. Graph-based fusion models further emphasized the importance of aligning architectural complexity with data characteristics, as their performance was sensitive to scaling choices and sample size, with some configurations failing to generalize effectively.

Late fusion approaches achieved stable but not superior performance, suggesting that aggregating modality-specific predictions may overlook fine-grained cross-modal interactions that are accessible through earlier integration. Nevertheless, this finding should be interpreted in light of the experimental setting. In more heterogeneous or partially observed datasets, decision-level strategies may offer increased robustness by allowing independent processing of each modality and limiting the propagation of modality-specific noise. More advanced late fusion strategies have been proposed in the literature, including approaches that optimize decision-level integration under criteria such as minimum probability of error or least mean square error. Therefore, the present results do not exclude the potential advantages of advanced late fusion strategies but rather indicate that their effectiveness is context-dependent. Exploring hybrid or adaptive combinations of early and late fusion represents a promising direction for future work.

These results also highlight the importance of considering trade-offs between model complexity, performance, computational cost, and interpretability. More complex architectures—such as attention-based models, variational autoencoders, and graph neural networks—typically involve a larger number of parameters and increased training time, which may lead to higher variance and reduced generalization when applied to moderately sized tabular datasets. In contrast, simpler feature-level approaches based on direct concatenation are characterized by lower complexity, more stable optimization, and reduced computational burden, enabling efficient learning of cross-modal interactions. Furthermore, simpler models facilitate interpretability by providing more transparent integration of multimodal features, whereas deeper architectures introduce additional layers of abstraction that may hinder interpretability without clear performance gains. Taken together, these findings suggest that increasing architectural complexity does not inherently guarantee better performance and that the optimal balance between complexity, efficiency, and interpretability should be guided by the characteristics of the data and the application context.

From a practical perspective, these findings have important implications for real-world applications. The proposed framework relies on tabular features derived from neuroimaging pipelines, such as FreeSurfer-based morphometric measures, together with cognitive assessments, both of which are routinely collected in research settings and are increasingly available in clinical practice. Notably, the best-performing approach—early feature-level fusion based on direct feature concatenation—proved to be not only accurate but also computationally efficient and straightforward to implement. Unlike more complex architectures, which often require extensive tuning and large sample sizes, early fusion approaches can be readily integrated into existing workflows using standard preprocessing techniques. This supports the practical applicability and scalability of the proposed approach, facilitating its potential translation from research to clinical settings. In addition, the entire benchmarking pipeline was implemented using the open-source *Fusilli* Python library, enabling full reproducibility of preprocessing, model configuration, and evaluation procedures, and supporting transparent comparison across datasets and tasks.

The use of sex classification as a benchmark was not intended to investigate biological sex per se but rather to provide a controlled and reproducible task in which performance differences primarily reflect methodological choices rather than target clinically meaningful variability ambiguously. Within this context, several limitations should be acknowledged.

First, the analysis was restricted to two modalities—cognitive measures and imaging-derived brain morphology—capturing only a subset of the multidimensional factors that contribute to brain organization. In addition, all experiments were conducted on a single healthy cohort within a relatively narrow age range, limiting generalizability to clinical populations or broader lifespan contexts.

Second, the study was designed under conditions of complete data availability, including only subjects with fully observed and synchronized modalities. While this ensures a fair comparison of fusion strategies, it may favor approaches based on joint feature learning. In more heterogeneous or partially observed datasets, decision-level strategies may provide increased robustness by allowing independent processing of each modality prior to integration. In addition, real-world multimodal medical data are often characterized by imbalance across modalities, including differences in data availability, quality, and sampling frequency. In the present study, this aspect was controlled by restricting the analysis to subjects with complete and synchronized modalities, enabling a fair comparison of fusion strategies. However, such conditions may favor early fusion approaches, which rely on joint feature representations, while late fusion strategies may provide increased robustness in the presence of missing, heterogeneous, or asynchronously acquired data. This highlights the importance of evaluating fusion methods under more realistic conditions, which will be addressed in future work.

An additional limitation concerns the demographic composition of the study cohort, which was restricted to participants who self-identified as White or Black within the HCP dataset. While this choice reduced population heterogeneity and potential confounding effects—thereby enabling a more controlled methodological comparison—it may introduce selection bias and limit generalizability to more diverse populations. Importantly, the primary aim of this study was methodological benchmarking rather than population-level inference, and this constraint does not affect the validity of the comparative evaluation. Nonetheless, future studies should extend this analysis to more diverse cohorts.

Finally, while this study focused on predictive performance, interpretability remains a critical requirement for neuroscience and clinical applications. Future research should extend the present framework in several directions, including the integration of additional modalities such as functional neuroimaging, genetic data, and longitudinal measures, as well as the application to disease-specific cohorts. The use of larger multicenter datasets and fully nested validation schemes will further improve robustness and reproducibility. In parallel, the integration of explainable artificial intelligence techniques will be essential to disentangle modality-specific contributions and enhance interpretability. The development of adaptive and data-driven fusion strategies, such as dynamic or modality-aware architectures, also represents a promising direction for optimizing multimodal integration in heterogeneous datasets.

Future research should further extend the proposed framework to clinically relevant prediction tasks, such as disease diagnosis and prognosis, and evaluate its robustness across more diverse and heterogeneous populations. In addition, investigating the impact of missing or partially observed modalities and exploring more advanced and adaptive fusion strategies will be essential to improve generalizability in real-world scenarios.

Taken together, these findings indicate that the effectiveness of multimodal deep learning in neuroscience depends critically on the joint selection of fusion strategy and preprocessing, rather than on model complexity alone. Through a systematic comparison of unimodal, early, intermediate, and late fusion approaches under controlled conditions, this study demonstrates that simple feature-level integration—when coupled with appropriate normalization—can outperform more elaborate fusion mechanisms. Although biological sex was used exclusively as a benchmarking variable, the proposed framework provides a generalizable methodological reference for evaluating multimodal fusion strategies and can be readily extended to clinically relevant applications, including biomarker discovery and disease characterization.

## Figures and Tables

**Table 1:** Hyperparameter settings for the evaluated data fusion models.

Hyperparameter	Attention and Activation	Attention Weighted GNN	Edge Correlation GNN
Attention Reduction Ratio	32	—	—
Drop Out Probability	—	0.1	0.1
Threshold	—	—	0.75
Patience	—	250	—

— = default value.

**Table 2:** Demographics of the HCP Young Adult Cohort.

Data	Male (n = 337)	Female (n = 410)
Age (in years)	28.2 ± 3.5	29.9 ± 3.4
Education level (in years)	15.1 ± 1.7	15.3 ± 1.7
MMSE ^1^	29.1 ± 1.0	29.1 ± 0.9

^1^ MMSE = Mini-Mental State Examination. Data are expressed as mean ± standard deviation. n = number of subjects (sample size).

**Table 3:** Classification performance (AUC–ROC/Accuracy) of unimodal and multimodal fusion models across different feature scaling strategies.

Data Fusion Model	Standard Scaling	Min–Max Scaling	Robust Scaling
Performance	AUC-ROC (CI)	AUC-ROC (CI)	AUC-ROC (CI)
	ACC (95% CI)	ACC (95% CI)	ACC (95% CI)
Tab1-Uni	0.77 (0.75–0.81)	0.79 (0.77–0.81)	0.78 (0.76–0.81)
	0.70 (0.68–0.71)	0.70 (0.69–0.71)	0.72 (0.70–0.75)
Tab2-Uni	0.91 (0.89–0.93)	0.93 (0.93–0.94)	0.91 (0.90–0.93)
	0.84 (0.82–0.86)	0.83 (0.78–0.88)	0.84 (0.82–0.86)
Concat-TabData	0.96 (0.95–0.96)	0.94 (0.92–0.98)	0.95 (0.94–0.95)
	0.88 (0.86–0.91)	0.85 (0.82–0.90)	0.88 (0.88–0.88)
Concat-TabFeat	0.90 (0.89–0.92)	0.93 (0.91–0.95)	0.92 (0.90–0.93)
	0.84 (0.83–0.86)	0.86 (0.83–0.89)	0.85 (0.84–0.86)
Channel-MultiNet	0.86 (0.86–0.89)	0.60 (0.50–0.80)	0.86 (0.85–0.88)
	0.87 (0.85–0.89)	0.58 (0.45–0.79)	0.85 (0.84–0.86)
Tab-CrossMHA	0.92 (0.89–0.92)	0.94 (0.93–0.96)	0.92 (0.91–0.94)
	0.84 (0.82–0.88)	0.87 (0.86–0.88)	0.86 (0.85–0.88)
AF-TabSelfAtt	0.79 (0.77–0.8)8	0.77 (0.75–0.79)	0.80 (0.79–0.81)
	0.70 (0.69–0.71)	0.61 (0.44–0.71)	0.71 (0.67–0.75)
MCVAE Tab	0.92 (0.90–0.93)	0.87 (0.84–0.90)	0.91 (0.90–0.92)
	0.83 (0.81–0.86)	0.73 (0.68–0.79)	0.82 (0.80–0.84)
EdgeCorr-GNN	0.92 (0.90–0.94)	0.57 (0.51–0.65)	0.92 (0.89–0.96)
	0.84 (0.82–0.86)	0.45 (0.40–0.48)	0.83 (0.80–0.86)
AttWeighted-GNN	0.52 (0.49–0.56)	0.50 (0.46–0.53)	0.51 (0.50–0.53)
	0.47 (0.46–0.53)	0.45 (0.43–0.46)	0.46 (0.43–0.49)
Tab-Decision	0.91 (0.89–0.92)	0.93 (0.92–0.94)	0.91 (0.90–0.94)
	0.85 (0.84–0.86)	0.80 (0.80–0.81)	0.83 (0.79–0.88)

Abbreviations: ACC = Accuracy; Tab1-Uni = Tabular1 Unimodal; Tab2-Uni = Tabular2 Unimodal; Concat-TabData = Concatenating Tabular Data; Concat-TabFeat = Concatenating Tabular Feature Maps; Channel-MultiNet = Channel-Wise MultiNet; Tab-CrossMHA = Tabular Crossmodal Multi-Head Attention; AF-TabSelfAtt = Activation-Function and Tabular Self-Attention; MCVAE Tab = MCVAE Tabular; EdgeCorr-GNN = Edge Correlation GNN; AttWeight-GNN = Attention-Weighted GNN; Tab-Decision = Tabular Decision.

## Data Availability

The data analyzed in this study are publicly available from the Human Connectome Project (HCP) Young Adult dataset (WU–Minn Consortium) and can be accessed through ConnectomeDB (https://db.humanconnectome.org) upon registration and agreement to the data use terms. No new data were generated during the study. The code used to implement the multimodal fusion models is available from the corresponding author upon reasonable request.

## Supplementary Materials

The following supporting information can be downloaded at: https://www.mdpi.com/article/10.3390/brainsci16040405/s1, Table S1. Classification performance of unimodal and multimodal fusion models across different feature scaling strategies; Figure S1. Receiver operating characteristic (ROC) and precision–recall (PR) curves for the best-performing models under Min–Max and Robust Scaling; Figure S2. Receiver operating characteristic (ROC) curves in the low false-positive rate regime across preprocessing strategies.

## Abbreviations

The following abbreviations are used in this manuscript:

GNN	Graph Neural Network
HCP	Human Connectome Project
PSQI	Pittsburgh Sleep Quality Index
ER40	ER40—Emotion Recognition Task (Penn Emotion Recognition Test)
ASR	Adult Self-Report
MMSE	Mini-Mental State Examination
IQR	InterQuartile Range
AUC-ROC	Area Under the Receiver Operating Characteristic Curve
PR	Precision-Recall
MCVAE	Multi-Channel Variational Autoencoder
MADDi	Multimodal Attention Deep Learning Framework
