# Supplementary material for: Benchmarking Multimodal Deep Fusion Strategies for Heterogeneous Neuroimaging and Cognitive Data Using a Controlled Sex Classification Task
Source: Brain Sci. 2026 Apr 10;16(4):405. doi: 10.3390/brainsci16040405 (PMC13114584; doi:10.3390/brainsci16040405)
Supplement: Supplementary file 1 [file brainsci-16-00405-s001.zip › brainsci-4186634-supplementary.pdf]

Supplementary Material

Supplementary Table S1. Classification performance of unimodal and multimodal fusion models across different feature scaling strategies.

Data fusion model	Standard Scaling	Min-Max Scaling	Robust Scaling
Performance	Balanced ACC (95% CI)	Balanced ACC (95% CI)	Balanced ACC (95% CI)
Tab1-Uni	0.70 (0.68-0.72)	0.71 (0.71-0.72)	0.73 (0.70-0.76)
Tab2-Uni	0.84 (0.82-0.85)	0.84 (0.81-0.89)	0.84 (0.83-0.86)
Concat-TabData	0.88 (0.86-0.91)	0.85 (0.82-0.90)	0.88 (0.88-0.88)
Concat-TabFeat	0.84 (0.83-0.85)	0.86 (0.83-0.90)	0.85 (0.84-0.86)
Channel-MultiNet	0.87 (0.85-0.89)	0.59 (0.50-0.80)	0.85 (0.83-0.86)
Tab-CrossMHA	0.85 (0.82-0.88)	0.87 (0.86-0.89)	0.86 (0.85-0.88)
AF-TabSelfAtt	0.70 (0.69-0.72)	0.64 (0.50-0.71)	0.72 (0.68-0.72)
MCVAE Tab	0.83 (0.81-0.86)	0.75 (0.72-0.79)	0.83 (0.82-0.85)
EdgeCorr-GNN	0.85 (0.82-0.86)	0.50 (0.50-0.50)	0.84 (0.80-0.87)
AttWeighted-GNN	0.50 (0.46-0.54)	0.50 (0.49-0.50)	0.49 (0.47-0.51)
Tab- Decision	0.85 (0.84-0.86)	0.82 (0.80-0.82)	0.84 (0.82-0.88)

Abbreviations: ACC = Accuracy; Tab1-Uni = Tabular1 Unimodal; Tab2-Uni = Tabular2 Unimodal; Concat-TabData = Concatenating Tabular Data; Concat-TabFeat = Concatenating Tabular Feature Maps; Channel-MultiNet = Channel-Wise Multi Net; Tab-CrossMHA = Tabular Crossmodal Multi-Head Attention; AF-TabSelfAtt = Activation-Function and Tabular Self-Attention; MCVAE Tab = MCVAE Tabular; EdgeCorr-GNN = Edge Correlation GNN; AttWeight-GNN = Attention-Weighted GNN; Tab-Decision = Tabular Decision;

Data fusion model	Standard Scaling	Min-Max Scaling	Robust Scaling
Performance	F1-score (95% CI)	F1-score (95% CI)	F1-score (95% CI)
Tab1-Uni	0.70 (0.68-0.73)	0.72 (0.68-0.77)	0.74 (0.71-0.76)
Tab2-Uni	0.85 (0.82-0.88)	0.82 (0.78-0.89)	0.85 (0.83-0.87)
Concat-TabData	0.90 (0.89-0.91)	0.85 (0.83-0.90)	0.89 (0.88-0.89)
Concat-TabFeat	0.85 (0.82-0.88)	0.86 (0.80-0.90)	0.86 (0.85-0.87)
Channel-MultiNet	0.88 (0.86-0.90)	0.50 (0.50-0.83)	0.87 (0.87-0.87)
Tab-CrossMHA	0.86 (0.83-0.90)	0.88 (0.87-0.89)	0.87 (0.85-0.88)
AF-TabSelfAtt	0.71 (0.70-0.73)	0.47 (0.47-0.71)	0.71 (0.65-0.77)
MCVAE Tab	0.85 (0.82-0.87)	0.70 (0.63-0.76)	0.83 (0.82-0.84)
EdgeCorr-GNN	0.85 (0.82-0.87)	0.72 (0.69-0.75)	0.84 (0.80-0.86)
AttWeighted-GNN	0.46 (0.39-0.53)	0.41 (0.41-0.43)	0.32 (0.12-0.53)
Tab- Decision	0.85 (0.84-0.87)	0.79 (0.77-0.80)	0.83 (0.79-0.88)

Abbreviations: Tab1-Un i= Tabular1 Unimodal; Tab2-Uni = Tabular2 Unimodal; Concat-TabData = Concatenating Tabular Data; Concat-TabFeat = Concatenating Tabular Feature Maps; Channel-MultiNet = Channel-Wise Multi Net; Tab-CrossMHA = Tabular Crossmodal Multi-Head Attention; AF-TabSelfAtt = Activation-Function and Tabular Self-Attention; MCVAE Tab = MCVAE Tabular; EdgeCorr-GNN = Edge Correlation GNN; AttWeight-GNN = Attention-Weighted GNN; Tab-Decision = Tabular Decision;

Data fusion model	Standard Scaling	Min-Max Scaling	Robust Scaling
**Performance**	Cohen's kappa (95% CI)	Cohen's kappa (95% CI)	Cohen's kappa (95% CI)
Tab1-Uni	0.40 (0.36-0.43)	0.41 (0.41-0.41)	0.45 (0.39-0.51)
Tab2-Uni	0.68 (0.65-0.70)	0.66 (0.58-0.77)	0.67 (0.65-0.71)
Concat-TabData	0.77 (0.73-0.82)	0.70 (0.64-0.79)	0.75 (0.75-0.75)
Concat-TabFeat	0.68 (0.65-0.71)	0.72 (0.66-0.78)	0.70 (0.68-0.71)
Channel-MultiNet	0.74 (0.69-077)	0.19 (0.18-0.56)	0.70 (0.67-0.73)
Tab-CrossMHA	0.68 (0.63-0.77)	0.74 (0.72-0.77)	0.72 (0.70-0.75)
AF-TabSelfAtt	0.40 (0.38-0.43)	0.27 (0.25-0.41)	0.42 (0.40-0.51)
MCVAE Tab	0.66 (0.62-0.72)	0.49 (0.40-0.58)	0.65 (0.61-0.68)
EdgeCorr-GNN	0.68 (0.63-0.71)	0.45 (0.42-0.55)	0.66 (0.60-0.73)
AttWeighted-GNN	0.60 (0.49-0.63)	0.60 (0.57-0.68)	0.51 (0.50-0.61)
Tab- Decision	0.69 (0.68-0.71)	0.61 (0.60-0.62)	0.68 (0.59-0.76)

Abbreviations: Tab1-Uni = Tabular1 Unimodal; Tab2-Uni = Tabular2 Unimodal; Concat-TabData = Concatenating Tabular Data; Concat-TabFeat = Concatenating Tabular Feature Maps; Channel-MultiNet = Channel-Wise Multi Net; Tab-CrossMHA = Tabular Crossmodal Multi-Head Attention; AF-TabSelfAtt = Activation-Function and Tabular Self-Attention; MCVAE Tab = MCVAE Tabular; EdgeCorr-GNN = Edge Correlation GNN; AttWeight-GNN = Attention-Weighted GNN; Tab-Decision = Tabular Decision;

Data fusion model	Standard Scaling	Min-Max Scaling	Robust Scaling
**Performance**	Average Precision (95% CI)	**Average Precision** (95% CI)	**Average Precision** (95% CI)
Tab1-Uni	0.79 (0.77-0.80)	0.82 (0.78-0.87)	0.78 (0.75-0.83)
Tab2-Uni	0.91 (0.87-0.93)	0.94 (0.93-0.95)	0.91 (0.89-0.94)
Concat-TabData	0.96 (0.96-0.97)	0.96 (0.93-0.97)	0.96 (0.95-0.97)
Concat-TabFeat	0.89 (0.83-0.92)	0.93 (0.90-0.95)	0.91 (0.89-0.94)
Channel-MultiNet	0.85 (0.83-0.87)	0.62 (0.51-0.78)	0.84 (0.83-0.85)
Tab-CrossMHA	0.91 (0.87-0.95)	0.95 (0.92-0.96)	0.93 (0.92-0.94)
AF-TabSelfAtt	0.82 (0.80-0.83)	0.81 (0.80-0.82)	0.82 (0.80-0.85)
MCVAE Tab	0.93 (0.91-0.95)	0.90 (0.87-0.91)	0.93 (0.93-0.93)
EdgeCorr-GNN	0.93 (0.91-0.96)	0.60 (0.55-0.64)	0.93 (0.90-0.97)
AttWeighted-GNN	0.57 (0.55-0.59)	0.54 (0.51-0.57)	0.56 (0.52 – 0.61)
Tab- Decision	0.91 (0.88-0.94)	0.94 (0.93-0.95)	0.92 (0.91-0.93)

Abbreviations: Tab1-Un i= Tabular1 Unimodal; Tab2-Uni = Tabular2 Unimodal; Concat-TabData = Concatenating Tabular Data; Concat-TabFeat = Concatenating Tabular Feature Maps; Channel-MultiNet = Channel-Wise Multi Net; Tab-CrossMHA = Tabular Crossmodal Multi-Head Attention; AF-TabSelfAtt = Activation-Function and Tabular Self-Attention; MCVAE Tab = MCVAE Tabular; EdgeCorr-GNN = Edge Correlation GNN; AttWeight-GNN = Attention-Weighted GNN; Tab-Decision = Tabular Decision;

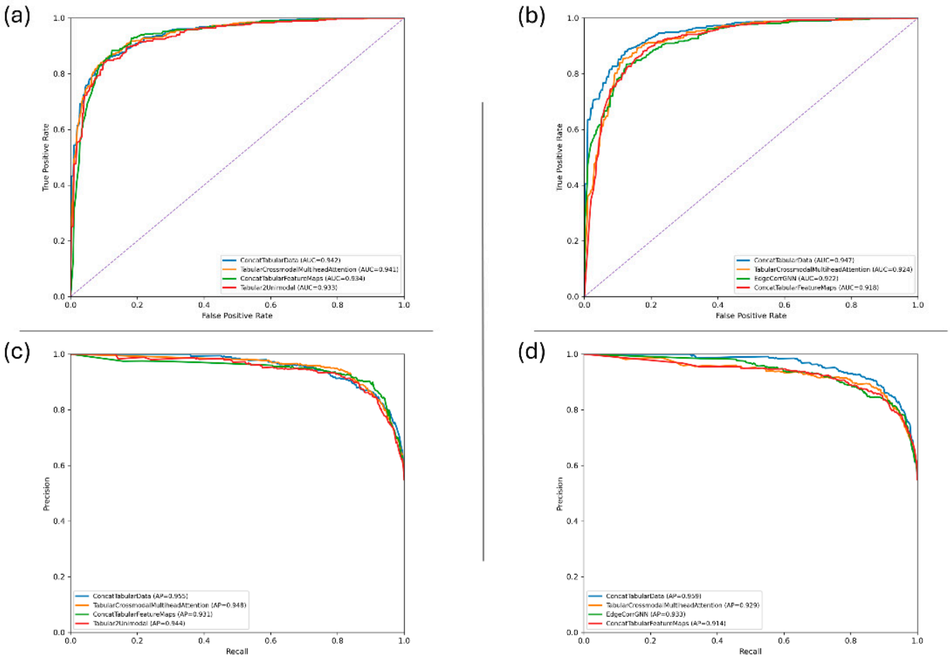

Supplementary Figure S1. Receiver operating characteristic (ROC) and precision–recall (PR) curves for the best-performing models under Min–Max and Robust Scaling. (a) ROC curves for models evaluated using Min–Max Scaling; (b) ROC curves for models evaluated using Robust Scaling; (c) PR curves for models evaluated using Min–Max Scaling; (d) PR curves for models evaluated using Robust Scaling. All curves are derived from predictions obtained through 3-fold cross-validation. AUC–ROC and average precision (AP) values are reported in the legend.

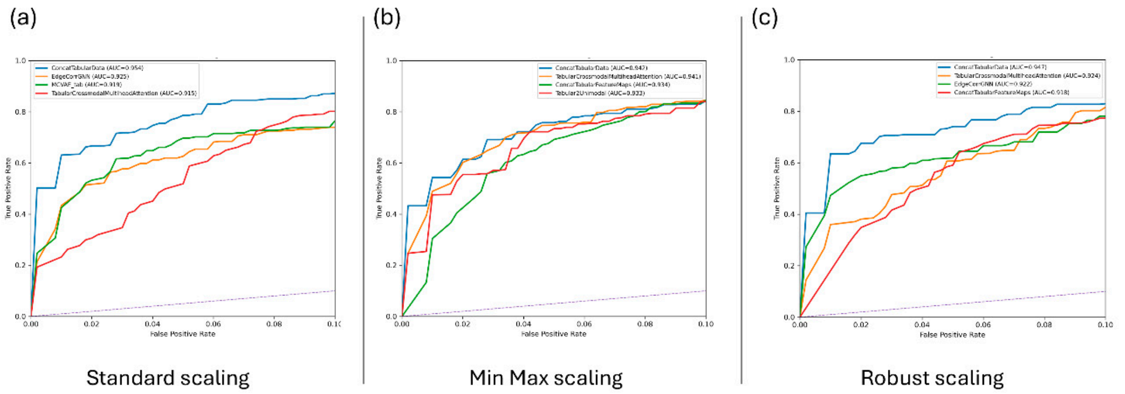

Supplementary Figure S2. Receiver operating characteristic (ROC) curves in the low false-positive rate regime across preprocessing strategies. (a) ROC curves for models evaluated using Standard Scaling; (b) ROC curves for models evaluated using Min–Max Scaling; (c) ROC curves for models evaluated using Robust Scaling. Curves are shown as zoomed-in views of the low false-positive rate region and are derived from predictions obtained through 3-fold cross-validation.
